# Do they really wash their hands? Prevalence estimates for personal hygiene behaviour during the COVID-19 pandemic based on indirect questions

**DOI:** 10.1186/s12889-020-10109-5

**Published:** 2021-01-04

**Authors:** Laura Mieth, Maike M. Mayer, Adrian Hoffmann, Axel Buchner, Raoul Bell

**Affiliations:** Department of Experimental Psychology, Heinrich Heine University Düsseldorf, Universitätsstrasse, 40225 Düsseldorf, Germany

**Keywords:** Coronavirus, COVID-19, Survey, Indirect questioning, Extended crosswise model, Randomised response technique

## Abstract

**Background:**

During the COVID-19 pandemic, billions of people have to change their behaviours to slow down the spreading of the virus. Protective measures include self-isolation, social (physical) distancing and compliance with personal hygiene rules, particularly regular and thorough hand washing. Prevalence estimates for the compliance with the COVID-19 measures are often based on direct self-reports. However, during a health crisis there is strong public pressure to comply with health and safety regulations so that people’s responding in direct self-reports may be seriously compromised by social desirability.

**Methods:**

In an online survey, an indirect questioning technique was used to test whether the prevalence of hygiene practices may be lower than in conventional surveys when confidentiality of responding is guaranteed. The Extended Crosswise Model is an indirect questioning technique that guarantees the confidentiality of responding. To the degree that direct self-reports are biased by social desirability, prevalence estimates of hygiene practices such as thorough hand washing based on the Extended Crosswise Model should be lower than those based on direct self-reports.

**Results:**

We analysed data of 1434 participants. In the direct questioning group 94.5% of the participants claimed to practice proper hand hygiene; in the indirect questioning group a significantly lower estimate of only 78.1% was observed.

**Conclusions:**

These results indicate that estimates of the degree of commitment to measures designed to counter the spread of the disease may be significantly inflated by social desirability in direct self-reports. Indirect questioning techniques with higher levels of confidentiality seem helpful in obtaining more realistic estimates of the degree to which people follow the recommended personal hygiene measures. More realistic estimates of compliance can help to inform and to adjust public information campaigns on COVID-19 hygiene recommendations.

**Supplementary Information:**

The online version contains supplementary material available at 10.1186/s12889-020-10109-5.

## Background

The global spread of COVID-19 (short for coronavirus disease 2019) was declared a pandemic by the World Health Organisation (WHO) on 11 March 2020 [29]. Due to its rapid progression, the COVID-19 pandemic puts a severe strain on health care systems. When too many people are infected at once, the mortality rate rises because not everyone can receive the necessary life-support measures [1]. Without an effective treatment, efforts to slow down the pandemic require billions of people to change their personal behaviours in their professional and private lives. Protective measures include self-isolation, social (physical) distancing and compliance with personal hygiene rules such as regular and thorough hand washing [30]. While governments can enforce restrictions to support some of the protective measures (e.g., by closing schools and by restricting public gatherings to reduce physical contacts among people), other measures (e.g., personal hygiene rules) are more difficult to enforce and control. Given that the success of the implemented protective measures requires the active participation and commitment of as many individuals as possible, it is necessary to evaluate the level of people’s commitment and then possibly to adjust public information campaigns. For that purpose, it is desirable to have valid information about the degree to which people comply with the recommended protective measures.

When people were asked directly about their behaviours, an overwhelming majority of the participants expressed compliance with the recommendations of political and health authorities during the first peak of the pandemic. For example, Mækelæ and colleagues reported that a percentage as high as 97.5% of the respondents of their multinational online community sample claimed to wash their hands as a protective action against COVID-19 [19]. However, to effectively reduce the spread of the virus during a worldwide health crisis, people have to accept many personal inconveniences for the greater public good. This social dilemma-type situation may create a moral obligation as well as strong public pressure to comply with health and safety regulations [27]. In consequence, direct self-reports may be compromised by social desirability. Adherence to “stay at home” directives can be inferred from mobility data retrieved from mobile phones. These data can be used to test whether, at group level, people’s mobility behaviour changes as a function of the social (physical) distancing guidelines [32]. However, for other types of protective measures such data are not publicly available because they concern intimate aspects of people’s personal lives. For these types of behaviours, valid prevalence estimates are difficult to obtain. Even when participants are assured of anonymity of their responding, some of the participants may still be concerned about confidentiality and may thus refuse to answer truthfully to sensitive questions when asked directly. This threatens the validity of prevalence estimates based on direct self-reports [25, 28].

To address this problem, *indirect* survey techniques have been developed (for an overview see for example [2, 14]). In indirect surveys based on the randomised response technique [28], random noise is added to the data to increase confidentiality. For example, participants may be presented with two sensitive questions that are mutually exclusive (A: “Have you *ever* used a cheat sheet during an exam?” and B: “Have you *never* used a cheat sheet during an exam?”). Instead of answering one of the questions directly, participants are instructed to spin a spinner unobserved by the interviewer. The spinner is marked in such a way that participants have to answer question A with probability *p* and question B with probability 1 – *p*. Given that the interviewer does not know which question is answered, the individual’s response to the question remains confidential but, given that the probability of the randomisation outcome is known, the prevalence of sensitive behaviours (such as using cheat sheets during exams) can be estimated at group level based on the distribution of the responses. Because this procedure guarantees the confidentiality of individual answers, participants are more inclined to admit to socially undesirable behaviours or attitudes than when asked directly [18]. However, the randomised response technique has been criticised for being rather difficult to explain and to implement due to the dependence on an external randomisation device. As an advancement, a simpler indirect survey technique, the Crosswise Model, has been developed [31]. This technique implies that participants are asked two questions simultaneously. One question is about a sensitive attribute for which the prevalence is of interest, the other is about a non-sensitive attribute with known prevalence. For instance, participants may be asked “Have you ever used a cheat sheet during an exam?” as well as “Is your mother’s date of birth in May, June or July?” (the prevalence of which can be estimated from official birth statistics). Participants are then instructed to respond to both questions simultaneously, choosing one of two response alternatives: “My answer is ‘yes’ to both questions or ‘no’ to both questions” or “My answer is ‘yes’ to one question and ‘no’ to the other question (irrespective of which one!)”. Neither of these response options reveals whether the participant carries the sensitive attribute so that individual responses remain confidential. Importantly, there is also no “safe” self-protective response option participants might choose to explicitly deny being a carrier of the sensitive attribute. An estimate for the prevalence of the sensitive attribute can still be obtained but only at group level, taking into account the prevalence of the non-sensitive attribute [31]. The Crosswise Model has led to higher and thus presumably more valid prevalence estimates than conventional direct questions for socially undesirable behaviours such as plagiarism [12] and tax evasion [13, 15], and to lower prevalence estimates for desirable attributes and behaviours such as trust [24] and dental hygiene [22]. What is more, the Crosswise Model has been shown to provide a more valid estimate of the prevalence of socially undesirable cheating behaviour than direct questioning in a strong validation study in which the prevalence of cheating behaviour was known [9].

In the present study we estimated the prevalence of compliance with personal hygiene rules (thorough hand washing) during the initial peak of the COVID-19 pandemic and tested whether the higher confidentiality of an indirect questioning method would lead to prevalence estimates of higher validity than the lower confidentiality of direct self-reports. We used the Extended Crosswise Model [6] that has been favourably evaluated in a recent experimental application [20]. The advantage of this simple but effective extension of the Crosswise Model is that it allows to detect certain types of non-adherence to the instructions without affecting estimation efficiency [6], as is explicated in the Results section below.

## Method

### Participants

Data were collected online between 26 and 30 March 2020, 2 weeks after COVID-19 was declared a pandemic by the WHO [29]. In Germany, severe restrictions on public life had been in place since 16 March 2020, including the closing down of all universities, schools and churches. With the number of deaths and infections increasing rapidly, recommendations for self-isolation, social (physical) distancing and personal hygiene were in full effect.

The sample was recruited online by spreading the survey link via widely used social media and messaging platforms such as Facebook and WhatsApp. The inclusion criteria (determined before data analysis) were that participants had to be (1) fluent in the German language, (2) of legal age (which is a requirement for being able to consent to the processing of one’s data), and (3) able to easily read the text on the screen and to complete the survey. In the instructions participants were asked to terminate the study if they were not able to complete the survey in privacy. Of 1595 participants who started the study 26 (1.6%) were screened out for not meeting the inclusion criteria and another 135 (8.5%) did not complete the survey.

We aimed at recruiting at least 1300 valid data sets based on an a-priori power analysis with G*Power [4], which showed that *N* ≥ 1300 participants were needed to detect small effects of at least *w* = 0.1 [3] when comparing the prevalence estimates between the direct and indirect questioning groups (*df* = 1) at a significance level of α = .05 with a statistical power of 1 – β = .95. At the end of the fifth day, data collection was terminated because our sample had surpassed this size. At that time, we had collected valid data sets of 1434 individuals (962 female, 469 male, 3 diverse) aged between 18 and 88 years (*M* = 35, *SD* = 15). The sample was well educated: 728 had a university degree, 574 had a university entrance qualification, 131 had a lower secondary school education and only one person had no formal school qualification. Further details on age, gender, and educational level by experimental group can be found in the Supplementary Material (Additional file 1). Participants were randomly assigned to the three experimental groups described below. The study was carried out in accordance with the Declaration of Helsinki.

### Material and procedure

The online survey was conducted using the software *SoSci Survey* [17]. All participants were asked to fill out the survey in privacy and declared their consent before starting the study. Then they were asked to answer (with “yes” or “no”) whether they met all inclusion criteria (“I am of legal age [at least 18 years old], I have good German language skills and I am able to easily read the text on the screen”). All participants were informed that they would be asked a question concerning their own behaviour during the time at which the protective measures against the spread of COVID-19 were in effect in Germany. Participants were randomly assigned to one of three experimental groups using the randomization (without replacement) function implemented in SoSci Survey. The 491 participants in the direct questioning group were asked to provide direct self-reports. Participants in the indirect questioning groups (471 participants in Extended Crosswise Model Group 1 and 472 participants in Extended Crosswise Model Group 2) were asked two questions simultaneously. Consistent with the Extended Crosswise Model proposed by Heck et al. [6], the only difference between the two indirect questioning groups was that the non-sensitive question (and, thus, the randomisation probability) differed between groups, as explained below.

#### Sensitive question

The sensitive behaviour “wash your hands regularly and sufficiently long (at least 20 seconds) with soap and water” was taken verbatim from a list of the five most important personal hygiene rules for protecting against COVID-19 provided by the German Federal Ministry of Health together with the German Federal Centre for Health Education [5].

The sensitive question was: “Do you wash your hands regularly and sufficiently long (at least 20 seconds) with soap and water?”

#### Direct questioning group

Participants in the direct questioning group received the following instructions:

Please think about your own behaviour since the protective measures against the coronavirus in Germany have been put into effect and answer the following question:

- Do you wash your hands regularly and sufficiently long (at least 20 seconds) with soap and water?

The question had to be answered with “yes” or “no”.

#### Indirect questioning groups

Participants in the indirect questioning groups received a detailed explanation of the Extended Crosswise Model technique. We explained that their response in the subsequent survey would remain confidential because they would give a response to two questions simultaneously so that it was impossible for us to know the answer to each individual question. The same example was used as in the present introduction (“Have you ever used a cheat sheet during an exam?”) to explain the procedure to the participants. Participants were assured that we did not know, and would not ask about, their mother’s birthday so that we could only infer the percentage of people showing the sensitive behaviour across all participants, but could not make inferences about their individual behaviour based on their response so that their answer to the sensitive question would remain confidential.

Participants in the Extended Crosswise Model Group 1 received the following instructions:

Please think about your own behaviour since the protective measures against the coronavirus in Germany have been put into effect and answer the following questions simultaneously:

1. Is your mother’s birthday in May, June or July?
2. Do you wash your hands regularly and sufficiently long (at least 20 seconds) with soap and water?

The question had to be responded to with either “My answer is ‘yes’ to both questions or ‘no’ to both questions” or “My answer is ‘yes’ to one question and ‘no’ to the other question (irrespective of which one!)”.

The instructions in the Extended Crosswise Model Group 2 were identical, but the non-sensitive question was: “1. Is your mother’s birthday in August, September, October, November, December, January, February, March or April?” so that the randomisation probability was complementary to the probability used in Extended Crosswise Model Group 1, thus meeting the requirements of the Extended Crosswise Model that allows to detect instruction non-adherence [6]. The questionnaire can also be found in the Supplementary Material (Additional file 2).

#### Demographic questionnaire

Next, participants were asked to report their age, gender and educational level. Only participants in the direct questioning group were also asked whether or not their mother’s birthday was in August, September, October, November, December, January, February, March or April. In that way we obtained a sample estimate for the prevalence of the non-sensitive attribute. Finally, all participants were thanked, debriefed and provided with a link to the current recommendations of the German health authorities for protecting against COVID-19.

## Results

**Fig. 1:**
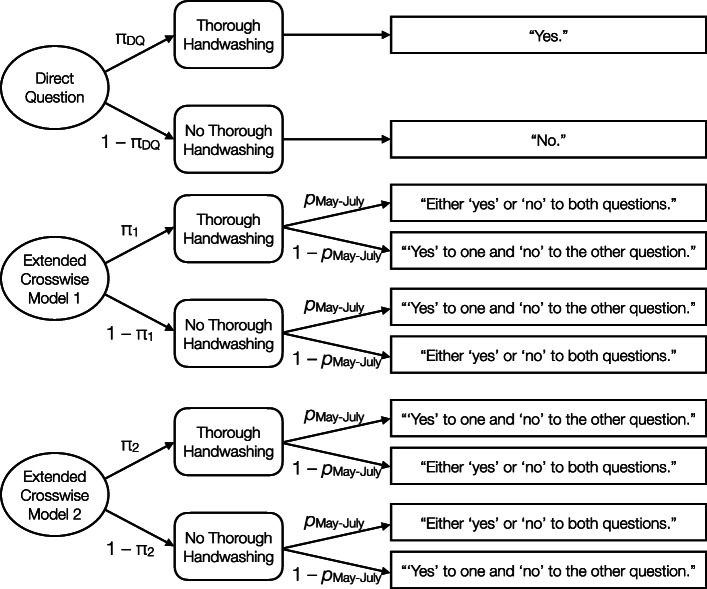
Multinomial Processing Tree Model. Combined multinomial processing tree model for the direct questioning group (upper tree) and for the two indirect questioning groups for the Extended Crosswise Model (two lower trees) adapted for the present question. The response options for the participants in the survey are depicted in the squares on the right. Parameter π _•_ represents the prevalence estimate for personal hygiene behaviour (thorough hand washing) during the COVID-19 pandemic (carriers of the sensitive attribute). Parameter *p*_May-July_ is the known prevalence of the answer to the non-sensitive question (the likelihood of the participant’s mother having been born in May, June or July), also referred to as the randomisation probability

The Extended Crosswise Model [6] illustrated in Fig. 1 was used to estimate the prevalence of the personal hygiene behaviour from the empirically observed response frequencies (Table 1). The software *multiTree* [21] was used to obtain maximum likelihood estimates [11] of the prevalence π in the three groups and to compare these estimates between the direct and indirect questioning conditions.

**Table 1:** Response frequencies in the direct and the two indirect questioning groups

Direct Question (*n* = 491)	
“Yes.”	464
“No.”	27
Extended Crosswise Model 1 (*n* = 471)	
“My answer is ‘yes’ to both questions or ‘no’ to both.”	172
“My answer is ‘yes’ to one question and ‘no’ to the other question (irrespective of which one!).”	299
Extended Crosswise Model 2 (*n* = 472)	
“My answer is ‘yes’ to both questions or ‘no’ to both.”	309
“My answer is ‘yes’ to one question and ‘no’ to the other question (irrespective of which one!).”	163

The upper tree represents the direct survey in which respondents either answered with probability π _DQ_ that they followed personal hygiene rules (thorough hand washing) to limit the spreading of COVID-19 or, with complementary probability 1 – π _DQ_, that they did not follow those rules. In the second and third tree illustrating the two Extended Crosswise Model groups, π _1_ and π _2_ again reflect whether the subjects followed the personal hygiene rules. In both trees, however, the sensitive question was presented together with the question about the non-sensitive attribute (the participant’s mother’s birthday). Parameter *p*_May-July_ thus represents the randomisation probability associated with the non-sensitive question, that is, the probability that the participant’s mother’s birthday is in May, June or July. In both indirect questioning groups (represented by Extended Crosswise Model 1 and Extended Crosswise Model 2 in Fig. 1), participants responded to the sensitive question and the randomisation question simultaneously, either by indicating that their answer was “either ‘yes’ or ‘no’ to both questions” or that their answer was “‘yes’ to one and ‘no’ to the other question”. The difference between the second and the third tree results from the complementary randomisation probabilities for the two different indirect questioning groups. Note that participants in the first indirect questioning group were asked whether their mother’s birthday was in May, June or July whereas participants in the second indirect questioning group were asked whether their mother’s birthday was in August, September, October, November, December, January, February, March or April. Therefore, the non-sensitive attribute was applied to participants with probability 1 – *p*_May-July_ in the second indirect questioning group. Thus, the response alternatives shown on the right side of the model have to be swapped in Extended Crosswise Model 2 compared to Extended Crosswise Model 1.

In the direct questioning group, we asked participants about their mother’s birthday which allowed us to conclude that the prevalence of the non-sensitive attribute in the present sample was 24.2%. Given that participants were randomly assigned to the groups, there is no reason to assume that this probability differed between the direct questioning group and the two indirect questioning groups. Accordingly, we set the randomisation parameter *p*_May-July_ to .242. However, note that the central conclusions derived from the following analysis would remain the same if the prevalence estimate of the non-sensitive attribute were based on official birth statistics in Germany from 1990, 2000 and 2010, published by the German Federal Agency for Statistics [23], according to which, on average, about 25.6% of all children were born in May, June or July.

In a first step, we tested whether the prevalence estimates for compliance with personal hygiene rules (π _•_) differed between the two indirect questioning groups, which would be indicative of deliberate non-adherence to the instructions, misunderstanding of the instructions or careless responding [6]. A baseline model incorporating the assumption that π did not differ between Extended Crosswise Model 1 and Extended Crosswise Model 2 groups (π _1_ = π _2_) fit the data well, *G*^2^(1) = 0.41, *p* = .524, suggesting that the obtained estimates were trustworthy and could be pooled across the two indirect questioning groups [6].

In the direct questioning group, π _DQ_ = 94.5% (*SE* = 1.0) of the participants stated that they complied with the hygiene rules concerning thorough hand washing. In the indirect questioning groups, the prevalence estimate was considerably lower with π _1&2_ = 78.1% (*SE* = 3.0). Restricting the prevalence parameters to be equal across groups (π _DQ_ = π _1&2_) significantly decreased the model fit, Δ*G*^2^(1) = 27.64, *p* < .001, indicating that the prevalence estimates in the direct and indirect questioning conditions were significantly different (|diff.| = 16.4%).

## Discussion

In the absence of an effective treatment, it is vitally important that people adopt behaviours minimising the risk of infection to reduce the spread of the COVID-19 pandemic. To adjust public awareness campaigns and to assess their effectiveness, obtaining valid information about people’s commitment to the measures against the spreading of COVID-19 is essential. Some health recommendations concern people’s private lives. Therefore, the degree to which people follow these recommendations cannot be assessed using publicly available data, leaving self-reports as the only source of information on whether people adopt responsible behaviours to prevent the spread of the disease. In the current study, we tested whether prevalence estimates based on direct questioning differed from those obtained with an indirect questioning technique. The Extended Crosswise Model [6] allows to estimate the prevalence of the sensitive behaviour at a group level but does not allow to draw any conclusions about an individual’s behaviour, thus guaranteeing a higher level of confidentiality than direct self-reports. Furthermore, this particular indirect questioning procedure does not offer a “safe” self-protective response option to which participants could resort [31], potentially further reducing the influence of social desirability bias and thereby increasing the validity of the obtained prevalence estimates.

The present results show that, when asked directly, an overwhelming majority of the respondents (94.5%) indicated to be committed to the recommendation of political and health authorities to wash their hands thoroughly, in line with previous self-reports on the acceptance of COVID-19 countermeasures during the first peak of the pandemic [19]. However, the prevalence estimate was substantially lower (78.1%) when it was based on an indirect question that guaranteed confidentiality, and the model-based comparison indicated that the difference between the prevalence estimates obtained with direct and indirect questioning (16.4%) was significant. This suggests that the prevalence estimate of the personal hygiene behaviour during the first peak of the COVID-19 pandemic derived from direct self-reports was inflated by social desirability. According to the “less is better” criterion [26], it can be assumed that in the indirect survey the influence of social desirability bias was successfully decreased so that substantially fewer people claimed to show the socially desirable behaviour (washing their hands thoroughly). The prevalence estimate for compliance with hygiene rules based on indirect questioning may therefore paint a more realistic picture than the estimate based on direct self-reports. It thus seems important to acknowledge a potential influence of social desirability bias when interpreting data on personal hygiene behaviours during the pandemic.

Indirect questioning techniques such as the Extended Crosswise Model may be helpful to arrive at more realistic prevalence estimates of sensitive information such as personal hygiene behaviours for which self-reports are the only source of information. However, there are also some limitations one should be aware of before applying this technique to epidemiological questions. First, the Crosswise Model technique [31] can only reveal more valid prevalence estimates for attitudes and behaviours respondents are explicitly aware of while implicit attitudes and automatic behaviours that escape introspection (e.g., touching one’s face) cannot be assessed [10, 25]. Second, randomisation techniques such as the Crosswise technique require larger samples than traditional direct surveys because there is a trade-off between confidentiality and efficiency [12]. The random error introduced by the randomisation question results in a greater sampling variance and lower statistical power so that larger samples are required to increase power. A large sample size is necessary also because parameter restrictions for the randomisation probability are only valid within a large group of participants so that large samples are necessary to arrive at valid conclusions about sensitive survey content. Third, participants have to understand that the randomisation technique guarantees the confidentiality of their answers [8, 16]. In the present study we examined a comparatively well-educated sample in which the ability to understand and to follow the instructions was presumably high. When applying the procedure to other populations or settings it may come at an advantage that the Extended Crosswise Model is able to detect some forms of instruction non-adherence such as the deliberate rejection of the truth, misunderstanding of the instructions and careless responding [6]. However, in these cases analyses with the Extended Crosswise Model can only reveal that the obtained data cannot be trusted and that the effort put into the questioning was in vain. A final, rather obvious limitation of indirect questioning techniques is that data can only be analysed at group level, making it impossible to gain insight into problematic or careless behaviours at an individual level. This also implies that the calculation of correlations with other variables in epidemiological research is rather complicated (for a useful statistical tool, however, see [7]).

## Conclusion

In summary, an indirect questioning technique that guaranteed a high degree of confidentiality led to a substantially lower estimate of personal hygiene behaviour (thorough hand washing) during the first peak of the COVID-19 pandemic than a conventional direct questioning approach. This result suggests that the prevalence estimate of thorough hand washing behaviour based on direct self-reports is inflated by social desirability and that prevalence of this desirable behaviour is in fact lower than what one would assume based on what people answer when asked directly.

## Supplementary Information

**Additional file 1.** Is a PDF containing age, gender and educational level separately for the three groups (Direct Question, Extended Crosswise Model 1, Extended Crosswise Model 2).

**Additional file 2.** Is a PDF with the questionnaire for the three groups in the survey (Direct Question, Extended Crosswise Model 1, Extended Crosswise Model 2).

## Data Availability

The questionnaire used in this study is presented in the Methods section and can be found in the Supplementary Material (Additional file 2). The data analysed in this study are included in Table 1.
