# Supplementary material for: Do they really wash their hands? Prevalence estimates for personal hygiene behaviour during the COVID-19 pandemic based on indirect questions
Source: BMC Public Health. 2021 Jan 4;21:12. doi: 10.1186/s12889-020-10109-5 (PMC7781177; doi:10.1186/s12889-020-10109-5)
Supplement: Supplementary file 1 — Additional file 1. Is a PDF containing age, gender and educational level separately for the three groups (Direct Question, Extended Crosswise Model 1, Extended Crosswise Model 2). [file 12889_2020_10109_MOESM1_ESM.pdf]

Age, Gender, Educational Level

Supplementary Material

Mieth, Mayer, Hoffmann, Buchner, & Bell: “Do they really wash their hands? Prevalence estimates for personal hygiene behaviour during the COVID-19 pandemic based on indirect questions“

The following table contains information on age, gender and educational level separately for the three groups (Direct Question, Extended Crosswise Model 1, Extended Crosswise Model 2).

	Direct Question	Extended Crosswise Model 1	Extended Crosswise Model 2
Age			
M (SD)	34 (15)	36 (15)	35 (16)
Gender			
Female	67 %	67 %	67 %
Male	33 %	33 %	32 %
Diverse	< 1 %	< 1%	< 1 %
Highest Educational Level			
University Degree	51 %	54 %	48 %
University Entrance Qualification	43 %	37 %	40 %
Lower Secondary School Education	7 %	9 %	12 %
No Formal School Education	0	0	< 1 %
