# Supplementary material for: Do they really wash their hands? Prevalence estimates for personal hygiene behaviour during the COVID-19 pandemic based on indirect questions
Source: BMC Public Health. 2021 Jan 4;21:12. doi: 10.1186/s12889-020-10109-5 (PMC7781177; doi:10.1186/s12889-020-10109-5)
Supplement: Supplementary file 2 — Additional file 2. Is a PDF with the questionnaire for the three groups in the survey (Direct Question, Extended Crosswise Model 1, Extended Crosswise Model 2). [file 12889_2020_10109_MOESM2_ESM.pdf]

Questionnaire

Supplementary Material

Mieth, Mayer, Hoffmann, Buchner, & Bell: "Do they really wash their hands? Prevalence estimates for personal hygiene behaviour during the COVID-19 pandemic based on indirect questions"

Participants in the *direct questioning group* received the following instructions.

> Please think about your own behaviour since the protective measures against the coronavirus in Germany have been put into effect and answer the following question:
>
> Do you wash your hands regularly and sufficiently long (at least 20 seconds) with soap and water?

The question had to be answered with "yes" or "no".

Participants in the *Extended Crosswise Model Group 1* received the following instructions:

> Please think about your own behaviour since the protective measures against the coronavirus in Germany have been put into effect and answer the following questions simultaneously:
>
> 1. Is your mother's birthday in May, June or July?
>
> 2. Do you wash your hands regularly and sufficiently long (at least 20 seconds) with soap and water?

The question had to be responded to with either "My answer is 'yes' to both questions or 'no' to both questions" or "My answer is 'yes' to one question and 'no' to the other question (irrespective of which one!)".

Participants in the *Extended Crosswise Model Group 2* received the following instructions:

> Please think about your own behaviour since the protective measures against the coronavirus in Germany have been put into effect and answer the following questions simultaneously:
>
> 1. Is your mother's birthday in August, September, October, November, December, January, February, March or April?
>
> 2. Do you wash your hands regularly and sufficiently long (at least 20 seconds) with soap and water?

The question had to be responded to with either "My answer is 'yes' to both questions or 'no' to both questions" or "My answer is 'yes' to one question and 'no' to the other question (irrespective of which one!)".
